# The characteristics of serious suicide attempters in Japanese adolescents- comparison study between adolescents and adults

**DOI:** 10.1186/1471-244X-12-191

**Published:** 2012-11-08

**Authors:** Yoshitaka Kawashima, Takao Ito, Ryuichiro Narishige, Takuya Saito, Yoshiro Okubo

**Affiliations:** 1 Department of Neuropsychiatry, Nippon Medical School, 1-1-5, Sendagi, Bunkyo-ku, Tokyo, 113-8602, Japan

**Keywords:** Suicide attempts, Adolescents, Age differences, Critical care medicine

## Abstract

**Background:**

Suicide is the leading cause of death among Japanese adolescents, and they may commit suicide differently from adults. However, there are few studies in medical-based data concerning adolescent patients seriously attempting suicide. We aimed to explore the characteristics of serious suicide attempts in Japanese adolescents, comparing them with those in adults.

**Methods:**

We investigated adolescents who seriously attempted suicide and were treated at the Critical Care Medical Center (CCMC) of Nippon Medical School Hospital between 2000 and 2010, and we compared them with adult suicide attempters treated during 2009. We retrospectively studied medical records and collected clinical data and socio-demographic factors, including age, sex, psychiatric symptoms or diagnosis, methods of suicide attempt, motives for suicide attempt, previous deliberate self-harm, previous psychiatric history, parent loss experience, and previous psychiatric history in the family.

**Results:**

Adolescent attempters were 15 males and 44 females, 13 to 18 years old (mean 16.39). Adult attempters were 37 males and 65 females, 19 to 79 years old (mean 39.45). In comparison to adult attempters, adolescent attempters were more frequently diagnosed with Borderline Personality Disorder (BPD), had more school problems and parent loss experience, but they had less financial problems. Gender differences between adolescents and adults were examined, and male adolescent attempters were found to be more frequently diagnosed with schizophrenia and had less financial problems than their adult counterparts, while female adolescent attempters were more frequently diagnosed with BPD, had more school problems and parent loss, but they had less previous psychiatric history than their adult counterparts.

**Conclusions:**

Our findings indicated that adolescent attempters were more frequently diagnosed with BPD and had more school problems and parent loss experience but had less financial problems. Additionally, in male adolescent attempters, identifying patients with schizophrenia seemed important, as it was their most frequent psychiatric diagnosis. For female adolescents, adequately assessing family function and interpersonal conflicts seemed important, as they were more often diagnosed with BPD and had more school and family problems.

## Background

The number of suicides in Japan has continued to exceed 30,000 every year, with the rate remaining at around 25.0 per 100,000 individuals since 1998, the highest rate among developed countries [1,2]. In adolescents aged 15–19, the suicide rate is 2.4 per 100,000 individuals [3], with suicide being the leading cause of death [4]. In Japan, individuals 15–24 years old are the most vulnerable group for suicide attempts and suicide mortality [5]. Suicide attempts in adolescents have been recognized as a major public health problem in not only Japan but also in other countries all over the world, because of their frequency, likelihood for recurrence, health care costs, and high risk for completed suicide [6]. In Japan, suicide behavior and suicide ideation among adolescents have been investigated in general community populations 15–24 years old [7-9], but there are few studies of suicide attempters among adolescents to be found in medical-based data.

Suicide risk has been reported to be associated with psychiatric disorders [10-12], adverse childhood experiences [13], and family history [14]. Suicide attempts and especially repetition of attempts are high-risk factors for subsequent suicide [15-17]. Previous literature has indicated that 10-50% of adolescent suicide attempters reattempted [18], and that about 11% of these committed suicide [19]. Additionally, among individuals under 25 years of age, both those who committed suicide and those making serious suicide attempts were in a similar group with the same risk factors [20]. Therefore, studies of serious suicide attempters are as important as psychological autopsy studies investigating complete suicide attempters in consideration of suicide prevention. In addition, a survey of suicide attempters based on direct interviews makes it possible for us to learn more about their detailed characteristics, such as socio-demographic factors and psychiatric symptoms before their attempts.

Being in a developmentally transitional stage, adolescents will differ from adults. For example, during adolescence, they struggle to achieve body mastery, control sexual and aggressive urges, gain independence from the family, find new and appealing sexual relationships, and achieve a sense of identity [21]. Adolescents start to rely less on parents for support and more on their peers as they grow older [22]. Also, adolescents differ from adults in terms of their typical financial status, medical conditions, occupational responsibilities, coping styles, social support networks, and the stressors to which they are commonly exposed [18]. Furthermore, suicidal behavior among adolescents occurs in different contexts from older individuals [23]. For example, adolescent suicidal behavior often occurs in the context of family conflict, including strivings for autonomy, in the context of academic and disciplinary difficulties, and in the consequence of disruptions in peer relationships that are important as youths get older. These have been described in previous literature, which pointed out the importance of the human lifespan in consideration of measures to combat suicide as well as interventions for mental health problems of adolescents, including suicidality, that are not developmentally tailored [24].

Therefore, in the present study, we explored the characteristics of serious suicide attempters among adolescents admitted to a critical care medical center in Japan, comparing them with adults.

## Methods

### Procedure

This study was carried out at the Critical Care Medical Center (CCMC) of Nippon Medical School Hospital, Tokyo. Since all patients at CCMC are in medically serious and potentially fatal condition, they require a high level emergency care. Approximately 2000 patients per year are admitted to CCMC, and about 100 of them are suicide attempters (excluding complete suicides) per year. Consultation-liaison psychiatrists have been assigned to provide psychiatric services to all suicide attempters admitted to CCMC [25]. Psychiatrists evaluate patients on admission and periodically during their CCMC stay to manage their psychiatric problems [26].

In this study, we defined adolescents as 18 years old or younger, and adults as 19 years old or older. Adolescent suicide attempters admitted to CCMC totaled 59 during the 11-year period between January 2000 and December 2010. In this study, we regarded adult suicide attempters as the control group for comparison with adolescents. Additionally, the period of the adults was fixed at one year, as the adolescent sample size was small. We selected the 1-year period of January-December 2009, in which the gender ratio between adolescents and adults was most similar within the past five years (between 2006 and 2010). Thus we included 102 adult suicide attempters from 2009 in our study.

We conducted a retrospective study of medical records containing information about primary care or any prior psychiatric treatment. We collected the clinical data and socio-demographic factors of all participants, including age, sex, psychiatric symptoms or diagnosis, methods of suicide attempts, motives for suicide attempts, previous deliberate self-harm, previous psychiatric history, parent loss experience by age 18, and previous psychiatric history in the family within a third degree of kinship.

In addition, we distinguished suicide attempts from non-suicidal self-injury without intent to die. We used definitions of suicide attempt based on the recent consensus on nomenclature for suicidology [27,28]. We defined suicide attempt as self-injurious behavior potentially resulting in fatality with at least some intent to end one’s own life. Suicide attempt also was determined on the basis of previous desire to die and a history of suicide attempts.

### Assessment of psychiatric disorders

Psychiatric diagnosis from medical records and information about any prior psychiatric treatment between January 2000 and December 2010 was based on DSM-IV and DSM-IV-TR by two or more experienced psychiatrists. In this study, psychiatric diagnosis with DSM-IV was remade according to DSM-IV-TR criteria. In case of disagreement with each other’s psychiatric diagnosis, a decision was reached by mutual consent after holding discussions.

### Statistical analyses

At the initial analysis, we examined the differences between adolescents and adults, using Pearson’s chi-square test and Fisher’s exact test for dichotomous and nominal variables. At the second analysis, males and females were analyzed separately because of the consistently significant gender differences in previous studies of adolescent suicide attempts [29]. We used a significance level of *p*<0.05 and two-sided probability. The SPSS version 16.0.2 statistical package (SPSS Inc., Chicago, IL., 2008) was used for the entire analysis.

### Ethics

This study was approved by the ethics committee of Nippon Medical School Hospital, and conforms to the provisions of the Declaration of Helsinki.

## Results

The demographic characteristics of all participants are presented in Table 1. The adolescents consisted of 15 males (25%) and 44 females (75%), aged 13 to 18, mean 16.39 years, and standard deviation (*SD*) 1.5 years. The number of adolescent suicide attempters increased with age. The adults consisted of 37 males (36%) and 65 females (64%), aged 19 to 79, mean 39.45 years, and standard deviation (*SD*) 13.2 years. As shown in Table 1, the number of female attempters was larger than that of the male attempters from adolescents to adults except for individuals 50–59 years old. There were no significant differences between the gender ratios of adolescents and adults.

**Table 1:** Demographic characteristics of all participants

	**Adolescents**		**Adults**	
	***N*=59 (%)**		***N*=102 (%)**	
**Gender**				
Male	15 (25)		37 (36)	
Female	44 (75)		65 (64)	
**Age, mean**	16.39		39.45	
**Age**	Males	Females	Males	Females
13	0 (0)	3 (100)	-	-
14	0 (0)	4 (100)	-	-
15	5 (42)	7 (58)	-	-
16	3 (43)	4 (57)	-	-
17	2 (14)	12 (86)	-	-
18	5 (26)	14 (74)	-	-
19	-	-	0 (0)	2 (100)
20-29	-	-	6 (22)	21 (78)
30-39	-	-	11 (41)	16 (59)
40-49	-	-	9 (39)	14 (61)
50-59	-	-	9 (60)	6 (40)
60≧	-	-	2 (25)	6 (75)

First we examined the differences between adolescents and adults (Table 2). Concerning psychiatric disorders, Borderline Personality Disorder (BPD) was seen more often among adolescents (χ^2^(1)=4.42; *p*<0.05). Regarding motives for suicide attempts, school problems were more frequent among adolescents (χ^2^(1)=20.87; *p*<0.001) and financial problems were more frequent among adults (χ^2^(1)=8.33; *p*<0.01). As for socio-demographic characteristics, adolescents had more parent loss experience (χ^2^(1)=10.26; *p*<0.05).

**Table 2:** Differences between adolescents and adults

	**Total**			**Male**			**Female**		
	**Adolescent**	**Adult**	***p***	**Adolescent**	**Adult**	***p***	**Adolescent**	**Adult**	***p***
	***N*=59 (%)**	***N*=102 (%)**		***N*=15 (%)**	***N=37 *(%)**		***N*=44 (%)**	***N=*65 (%)**	
**DSM-IV-TR**^**a**^									
Substance-Related Disorders	1 (2)	5 (5)	*n.s.*	0 (0)	4 (11)	*n.s.*	1 (2)	1 (2)	*n.s.*
Schizophrenia	8 (14)	14 (14)	*n.s.*	6 (40)	3 (8)	<0.05	2 (5)	11 (17)	*n.s.*
Mood Disorders	21 (36)	47 (46)	*n.s.*	3 (20)	14 (38)	*n.s.*	18 (41)	33 (51)	*n.s.*
Adjustment Disorders	6 (10)	15 (15)	*n.s.*	1 (7)	8 (22)	*n.s.*	5 (11)	7 (11)	*n.s.*
Anxiety Disorders	3 (5)	2 (2)	*n.s.*	0 (0)	0 (0)	*n.s.*	3 (7)	2 (3)	*n.s.*
Borderline Personality Disorder ^b^	16 (27)	14 (14)	<0.05	0 (0)	4 (11)	*n.s.*	16 (36)	10 (15)	<0.05
Dependent Personality Disorder	0 (0)	1 (1)	*n.s.*	0 (0)	0 (0)	*n.s.*	0 (0)	1 (2)	n.s.
Other	11 (19)	12 (12)	*n.s.*	5 (33)	5 (14)	*n.s.*	6 (14)	7 (11)	*n.s.*
Indeterminate	6 (10)	5 (5)	*n.s.*	1 (7)	4 (11)	*n.s.*	5 (11)	1 (2)	<0.05
**Method of suicide attempt**									
Poisoning	41 (69)	65 (64)	*n.s.*	7 (47)	19 (51)	*n.s.*	34 (77)	46 (71)	*n.s.*
Jumping from high place	16 (27)	18 (18)	*n.s.*	6 (40)	7 (19)	*n.s.*	10 (23)	11 (17)	*n.s.*
Cutting	1 (2)	11 (11)	*n.s.*	1 (7)	8 (22)	*n.s.*	0 (0)	3 (5)	*n.s.*
Drowning	1 (2)	1 (1)	*n.s.*	1 (7)	0 (0)	*n.s.*	0 (0)	1 (2)	*n.s.*
Carbon monoxide poisoning	0 (0)	2 (2)	*n.s.*	0 (0)	1 (3)	*n.s.*	0 (0)	1 (2)	*n.s.*
Self-burning	0 (0)	1 (1)	*n.s.*	0 (0)	0 (0)	*n.s.*	0 (0)	1 (2)	*n.s.*
Hanging	0 (0)	1 (1)	*n.s.*	0 (0)	0 (0)	*n.s.*	0 (0)	1 (2)	*n.s.*
Gas	0 (0)	3 (3)	*n.s.*	0 (0)	2 (5)	*n.s.*	0 (0)	1 (2)	*n.s.*
**Motives for suicide attempt**^**a,c**^									
Family problem	20 (34)	38 (37)	*n.s.*	2 (13)	8 (22)	*n.s.*	18 (41)	30 (46)	*n.s.*
Financial problem	2 (3)	20 (20)	<0.01	1 (7)	15 (41)	<0.05	1 (2)	5 (8)	*n.s.*
Work problem	2 (3)	12 (12)	*n.s.*	0 (0)	5 (14)	*n.s.*	2 (5)	7 (11)	*n.s.*
Love problem	11 (19)	15 (15)	*n.s.*	2 (13)	1 (3)	*n.s.*	9 (20)	14 (22)	*n.s.*
School problem	13 (22)	1 (1)	<0.001	1 (7)	1 (3)	*n.s.*	12 (27)	0 (0)	<0.001
Other problem	17 (29)	24 (24)	*n.s.*	7 (47)	15 (41)	*n.s.*	10 (23)	9 (14)	*n.s.*
Unclear	1 (2)	1 (1)	*n.s.*	1 (7)	1 (3)	*n.s.*	0 (0)	0 (0)	*n.s.*
**Sociodemographic characteristics**									
Previous deliberate self-harm	31 (53)	64 (63)	*n.s.*	5 (33)	16 (43)	*n.s.*	26 (59)	48 (74)	*n.s.*
Previous psychiatric history	36 (61)	75 (74)	*n.s.*	8 (53)	22 (59)	*n.s.*	28 (64)	53 (82)	<0.05
Parent loss experience	20 (34)	13 (13)	<0.05	4 (27)	5 (14)	*n.s.*	16 (36)	8 (12)	<0.01
Previous psychiatric history in family	22 (37)	36 (35)	*n.s.*	5 (33)	8 (22)	*n.s.*	17 (39)	28 (43)	*n.s.*

^a^ There were repetitions in DSM-IV-TR and motives for suicide attempt. Therefore, some percentage totals are more than 100.

^b^ Borderline Personality Disorders included borderline personality trait.

^c^ We excluded psychiatric symptoms as motives for suicide attempt. Therefore, some percentage totals are less than 100.

Then we examined the differences between adolescents and adults by gender (Table 2). For males, schizophrenia among psychiatric disorders was more often observed in adolescents (Fisher’s exact test; *p*<0.05) and financial problems among motives were more often seen in adults (Fisher’s exact test; *p*<0.05). For females, BPD (χ^2^(1)=6.36; *p*<0.05), indeterminate (Fisher’s exact test; *p*<0.05) among psychiatric disorders, school problems (Fisher’s exact test; *p*<0.001) among motives, and parent loss experience (χ^2^(1)=8.84; *p*<0.01) among socio-demographic characteristics were more often found in adolescents. Also, among socio-demographic characteristics, previous psychiatric history was more frequently noted in adults (χ^2^(1)=4.41; *p*<0.05).

## Discussion

This is the first study to examine the characteristics of serious suicide attempters among adolescents in Japan, focusing on psychiatric disorders, methods, motives and socio-demographic factors compared with adults.

We first examined the differences between 59 adolescent suicide attempters and 102 adults. The results revealed significant differences in BPD among psychiatric disorders, in financial problems and school problems among motives for suicide attempts, and in parent loss experience among socio-demographic characteristics.

As for the gender ratio among suicide attempters, the ratio of females was higher than that of males both in adolescents and adults. The subsequent gender-specific analysis revealed different characteristics between male adolescents and female adolescents, highlighting the fact that, if we compare adolescent suicide attempters with adult suicide attempters, we should also consider their characteristics according to gender.

### Psychiatric disorders

A previous study reported that 95% of suicide attempters met the DSM-IV criteria for either axis I or axis II psychiatric diagnosis or both [30]. In the present study, psychiatric disorders were present in 98% of female adults, 93% of male adolescents, and 89% of both male adults and female adolescents. Therefore, among not only adults but also adolescents, psychiatric disorders have a close relation with attempted suicide, and there may be psychiatric symptoms or social dysfunction with psychiatric disorders behind suicide.

Depressive disorders are consistently the most prevalent disorders among adolescent suicide victims [29]. In the present study, mood disorders were also most common among adolescents. However, according to the analysis by gender, schizophrenia was the most prevalent psychiatric disorder among male adolescents and was also seen more often in male adolescents than in male adults. The onset of schizophrenia is earlier in males than in females [31] and schizophrenia patients committing suicide are more often men and tend to be young [32]. According to a recent meta-analysis, 4.9% of patients with schizophrenia die of suicide, most of them soon after illness onset [33]. Additionally, those with schizophrenia tend to use highly lethal suicide methods [32]. The reason for male adolescents to have a high rate of schizophrenia in our study might be related to the gender difference at onset age of the condition. Additionally, as all participants in our study were in a medically serious and potentially fatal state, there may have been a selection bias. It is important that we identify male patients with schizophrenia as possible adolescent suicide attempters, because schizophrenia among adolescents increased the risk of suicide [34]. We also need to provide comprehensive initial treatment that improves adherence to early treatment via a psycho-education for patients and their families, as 2-11% of patients with schizophrenia attempt suicide at least once a year after starting treatment [35].

In females, BPD was more common among adolescents than adults. We especially focused on the diagnosis of personality disorders in adolescent participants, as adolescence is the developmental stage of personality. We investigated psychiatric symptoms in medical records from psychiatrist-conducted interviews of all participants or their parents as well as from information about any prior psychiatric treatments. Previous studies have reported that the frequency of BPD in female adolescents who committed suicide was 26% [36] and that it was 33% in individuals 15–29 years old [37]. The result of the present study, that BPD was the second most common diagnosis for female adolescents, supported previous studies reporting that BPD was relatively frequently associated with suicide attempts and completed suicides among adolescents. Therefore, it is important to assess interpersonal relationships and emotional control of female adolescent suicide attempters with BPD. Likewise, it is necessary to examine possible developmental histories that might affect suicide attempts, focusing on the development of attachment, mother-child relationship problems, and traumatic episodes in childhood strongly associated with BPD. Indeterminate for psychiatric disorders also was more often seen among adolescents than adults. Some female adolescents may attempt suicide without any psychiatric symptoms and prior indications of emotional or behavioral problems. It has been pointed out that adolescents may be more impulsive than adults, and they may tend to focus on proximal consequences of behavior [38,39]. Therefore, we have to pay attention to impulses among female adolescents.

### Motives for suicide attempts and socio-demographic characteristics

As for motives, family problems had the highest incidence in both male and female adolescents. The relation between family functioning and adolescent suicide attempts has been explored in many lines of research [18,23]. Our findings supported those previous studies. On the other hand, we found that adolescents, and particularly females, had more parent loss experience and school problems. This finding may indicate that female adolescents are more vulnerable to stresses in the immediate environment and interpersonal relationships than male adolescents and adults. Therefore, in regard to female adolescents, it is important to adequately assess family functioning and interpersonal conflict, and furthermore, to reach out to the patients and their families.

Male adults had significantly more financial problems than male adolescents, while there was no difference between females. A previous study in Japan [40] reported that unemployment is an important suicidal risk factor in males. They may suffer more severe effects from failures on the job or struggles to survive from such as debts. For male adult suicide attempters with financial problems, they should be paid close attention, and adequate intervention with a view to solving their financial problems should be undertaken.

In terms of previous psychiatric history, female adults had significantly more than female adolescents, while there was no difference in males. This may indicate that the frequency of psychiatric care is greater in females than males and that the tendency becomes stronger with increasing age. Still, despite receiving psychiatric care in the past, more than half of all participants attempted suicide. If suicide attempters discontinue their psychiatric treatment, it is vital that their treatment be re-established. Even if they are undergoing psychiatric treatment, they may have poor medication adherence, severe psychiatric symptoms, and psychosocial impairments. Therefore, for the prevention of subsequent suicide attempts, we have to determine their psychosocial issues and remedy psychosocial dysfunctions in parallel with improving adherence and psychiatric symptoms as part of their psychiatric treatment.

### Limitations

This study has several limitations. First, it used a relatively small sample size, meaning that our results can only be generalized with caution. However, this study sample does present meaningful data, in consideration of measures to combat suicide, because all participants were serious suicide attempters with characteristics similar to complete suicide attempters. Another limitation is that the study period was different between adolescents and adults, and we could not control the differences between the time periods. A third limitation is that the psychiatric diagnoses of all participants were reached without structured interviews and assessment tools. It is difficult for psychiatrists to conduct structured and systematic interviews for suicide attempters because of the shorter hospitalization and temporal constraints in CCMC. We did our best to enhance the reliability of the psychiatric diagnoses, holding discussions in the case of disagreement with the psychiatric diagnoses. However, there may be an issue that the diagnoses in the present work are more studied, as they were based more on mental status records than diagnoses that are obtained by direct and structured questionnaires.

We did not divide adolescents or adults into subgroups according to age, such as younger adolescents or elderly adults, as our small sample size made it difficult to compare the different age generations in detail. Thus, in the present study, we just aimed to explore the robust differences between adolescents and adults. However, in a future study based on a larger study population we will have to investigate the differences among the various age groups from adolescents to adults. Moreover, it will be necessary to conduct a prospective longitudinal study by means of structured assessment tools and develop interventions tailored to adolescent characteristics.

## Conclusion

Our present findings indicate that adolescent attempters were more frequently diagnosed with BPD and had more school problems and parent loss experience but had less financial problems. Additionally, by gender, we found the importance of identifying patients with schizophrenia especially among male adolescents, because they are more commonly diagnosed with this disorder. Furthermore, this study has revealed that female adolescents more often receive a diagnosis of BPD, and they have more parent loss experience and school problems, indicating the importance of adequately assessing family functioning and interpersonal conflicts for female adolescents.

## Abbreviations

CCMC: Critical Care Medical Center; DSM: Diagnostic and Statistical Manual of Mental Disorders; BPD: Borderline Personality Disorder.

## Competing interests

The authors declare that they have no competing interests.

## Authors’ contributions

All authors contributed to study design. YK, TI, RN contributed to data collection. YK, RN, TS, YO wrote the analysis plan. YK conducted the statistical analysis and wrote the first draft of the manuscript. TI, RN, TS, YO discussed the ideas in the manuscript and contributed to its preparation. All authors read and approved the final manuscript.

## Pre-publication history

The pre-publication history for this paper can be accessed here:

http://www.biomedcentral.com/1471-244X/12/191/prepub
